# *COMT* and prenatal maternal smoking in associations with conduct problems and crime: the Pelotas 1993 birth cohort study

**DOI:** 10.1038/srep29900

**Published:** 2016-07-18

**Authors:** Angélica Salatino-Oliveira, Joseph Murray, Christian Kieling, Júlia Pasqualini Genro, Guilherme Polanczyk, Luciana Anselmi, Fernando Wehrmeister, Fernando C. de Barros, Ana Maria Baptista Menezes, Luis Augusto Rohde, Mara Helena Hutz

**Affiliations:** 1 Department of Genetics, Universidade Federal do Rio Grande do Sul, Porto Alegre, Rio Grande do Sul, Brazil; 2 Department of Psychiatry, University of Cambridge, United Kingdom; 3 Postgraduate Program in Epidemiology, Universidade Federal de Pelotas, Pelotas, Rio Grande do Sul, Brazil; 4 Division of Child and Adolescent Psychiatry, Hospital de Clínicas de Porto Alegre, Porto Alegre, Rio Grande do Sul, Brazil; 5 Department of Psychiatry, Universidade de São Paulo, São Paulo, Brazil; 6 Institute for Developmental Psychiatry for Children and Adolescents; Brazil; 7 Graduate Program in Health and Behavior, Universidade Católica de Pelotas, Pelotas, Rio Grande do Sul, Brazil

## Abstract

Conduct problems in childhood and adolescence are significant precursors of crime and violence in young adulthood. The purpose of the current study is to test the interaction between prenatal maternal smoking and *COMT Val*^*158*^*Met* in conduct problems and crime in the 1993 Pelotas Birth Cohort Study. Conduct problems were assessed through the parent version of the Strengths and Difficulties Questionnaire at ages 11 and 15 years. A translated version of a confidential self-report questionnaire was used to collect criminal data at 18 years of age. Negative binomial regression analyses showed an association between prenatal maternal smoking and SDQ conduct problem scores (IRR = 1.24; 95% CI: 1.14–1.34; p < 0.001) at 11 years of age. However, no evidence was found for an association between *COMT* genotypes and conduct scores or for an interaction between maternal smoking and this gene in predicting conduct problems. Very similar results were obtained using the 15 years conduct scores and crime measure at age 18. Prenatal maternal smoking was associated with crime (IRR = 1.28; 95% CI: 1.09–1.48; p = 0.002) but neither *COMT* genotypes nor the possible interaction between gene and maternal smoking were significantly associated with crime. Replications of GxE findings across different social contexts are critical for testing the robustness of findings.

Conduct problems refer to antisocial behaviors typical of disruptive behaviors disorders (DBD) which are conditions involving difficulties in self-control. DBD symptoms are related to behaviors that violate the rights of others and/or that bring the person into conflict with societal norms or authority figures^1^. Notably, the presence of conduct problems in childhood and adolescence are significant predictors of crime and violence in young adulthood^2^,^3^.

Gene x environment interaction (GxE) research in psychiatry has been identified as having great potential to uncover the etiology of mental disorders^4^. Pioneering studies in this area, especially using candidate GxE approaches, have had a large impact on recent psychiatric literature^5^,^6^,^7^. However, positive GxE findings have a low rate of replicability, causing considerable doubt and debates regarding their validity^4^,^8^,^9^.

One of the potential candidates for GxE studies in conduct problems is the catechol-O-methyltransferase (*COMT*) gene which encodes a key modulator of dopamine and norepinephrine pathways. The COMT enzyme breaks down catecholamines, moderating extracellular levels of these neurotransmitters mainly in the prefrontal cortex (PFC)^10^,^11^. The *Val*^*158*^*Met* polymorphism is a common functional single nucleotide polymorphism (SNP). The *Val* variant determines higher COMT activity in the PFC compared to the *Met* variant^12^,^13^. COMT seems to play a role in cerebral areas which modulate self-regulation and expression of negative emotions, affecting antisocial behavior and criminal involvement^14^. These neuropsychological problems could interact cumulatively with negative environments across development, resulting in persistent antisocial behavior through the life-course^2^. Presumably because of the relevance of COMT activity for PFC functionality, the *Val*^*158*^*Met* SNP has been associated with poorer executive functions, childhood disruptive disorders, antisocial behavior, aggression and attention-deficit hyperactivity disorder (ADHD)^15^,^16^,^17^,^18^,^19^,^20^,^21^. In a recent review, the *COMT* gene was highlighted as one of the four most studied genes related to dopamine and serotonin pathways in association with aggression-related phenotypes^21^. Recent GxE studies have shown that *COMT* genotypes may modify the sensivity to environments that confer either risk or protection for aggressive behavior^22^,^23^,^24^.

Three studies have examined the effect of the interaction between prenatal risk factors and *COMT Val*^*158*^*Met* polymorphism in antisocial behaviors^7^,^8^,^25^. Based on results linking both PFC and prenatal adversity with childhood-onset antisocial behavior, Thapar *et al*.^7^ uncovered a significant interaction between *COMT* genotype and lower birth weight in relation to conduct symptoms in a sample of children with ADHD; however this result was not replicated by Sengupta *et al*.^8^. Brennan *et al*.^25^ found that individuals with *Val/Val* genotype whose mothers smoked during pregnancy had an increased risk for aggressive behavior outcomes in adolescence and young adulthood. Nevertheless, as emphasized by the authors, replication studies are necessary.

The prenatal exposure to cigarette smoke is a potential risk factor for several adverse outcomes in delivery and across the life cycle of the offspring, including low birth weight, conduct problems, psychopathy and criminality^26^,^27^,^28^,^29^. Sibling-comparison studies showed that associations between prenatal maternal smoking and delivery outcomes persisted when discordant siblings were compared, consistent with causal interpretations^27^. On the other hand, associations of conduct problems and crime with maternal smoking during pregnancy were also explained by genetic factors^26^,^27^,^30^,^31^,^32^,^33^,^34^,^35^.

The GxE studies which have analyzed the *COMT* gene and antisocial behaviors, as well as the vast majority of other GxE investigations, have been performed in high-income countries. Considering the higher levels of antisocial behaviors such as violence in low- and middle-income countries (LMIC), such as Brazil, research performed in cohorts from LMIC provides an important opportunity to test the robustness of risk factors associated with mental health in other settings^3^,^36^,^37^. As such, the aim of the present study was to test the interaction between maternal smoking during pregnancy and the *COMT Val*^*158*^*Met* polymorphism in relation to conduct problems and criminal behavior.

## Results

A total of 4,101 adolescents provided saliva DNA samples at the age 15 visit. 4,095 individuals were successfully genotyped for the *Val*^*158*^*Met* polymorphism. The allele frequencies observed were 0.57 and 0.43 for the *Val* and *Met* alleles, respectively. The genotype distributions were consistent with Hardy–Weinberg equilibrium (χ^2^ = 3.098; df = 1; p = 0.08); 33.6% of individuals were *Val*/*Val* homozygous, 47.6% were heterozygous and 18.9% were *Met*/*Met* homozygous. Almost half of the sample were boys (48.9%) and around two thirds (63.7%) self-identified as having white skin color. The education of most mothers was between five and eight years of school attendance (43.6%) and the mean family monthly income was 4.2 minimum wages. About 5% of mothers reported having used alcohol and 33% reported smoking during pregnancy. For 30.8% of the cases, the mother was screened positive for psychopathology when children were aged 11 years. SDQ conduct scores decreased from 2.49 at age 11 to 2.29 at age 15. About 17% of individuals self-reported committing a crime in the previous 12 months at age 18/19. Detailed demographic and clinical characteristics of the sample according to genotype groups are presented in Table 1.

Negative binomial regression analyses showed main effect of maternal smoking during pregnancy in SDQ conduct scores at age 11 (IRR = 1.24; 95% CI: 1.14–1.34; p < 0.001) (Table 2). However, no evidence was found for an association between *COMT* genotypes and SDQ conduct scores (p = 0.932) (Table 2). Also, the interaction analysis did not show a significant interaction effect of maternal smoking and *COMT* gene in SDQ conduct scores at age 11 (p = 0.737; Supplementary Table S1). Very similar results were obtained in analyses of the age 15 SDQ conduct scores (Table 2 and Supplementary Table S1).

There was evidence of association between prenatal maternal smoking and crime (number of types of offences) at 18 years of age (IRR = 1.28; 95% CI: 1.09–1.48; p = 0.002) (Table 2). Nevertheless, *COMT* genotypes were not associated with crime (p = 0.282) (Table 2), and there was not a significant interaction between this gene and maternal smoking during pregnancy in predicting crime at age 18 (p = 0.196; Supplementary Table S1). Very similar results were found in logistic regression analysis using a dichotomous crime outcome (maternal smoking during pregnancy: OR = 1.26, 95% CI: 1.02–1.55, *P* = 0.032; *COMT* genotypes and the interaction term: p > 0.168 for both).

## Discussion

GxE research in psychiatric epidemiology needs to answer whether and how genetic risks are environmentally dependent^38^,^39^. Genetic variants that are already established as risk factors for mental disorders are investigated under varying environmental situations or vice versa. In this context, the study of an interaction effect between the *COMT* gene and environmental factors on antisocial and aggressive symptoms might be relevant, since this gene has been associated with disruptive disorders and antisocial behaviors^13^,^15^,^17^. Maternal smoking during pregnancy is considered an important risk factor for a range of adverse health outcomes in offspring, including conduct problems and criminal behavior^26^,^30^,^31^,^33^,^35^,^40^,^41^. Therefore, this study evaluated the possible interaction effect between the *COMT Val*^*158*^*Met* polymorphism and prenatal maternal smoking in conduct problems and crime. A large population-based birth cohort in a middle-income setting was used to attempt replication. Maternal tobacco use during gestation was associated with higher levels of conduct problems at ages 11 and 15 and crimes committed in late adolescence in the current study. However, neither *COMT Val*^*158*^*Met* polymorphism effect nor interactions between this polymorphism and prenatal maternal smoking were observed.

Skepticism and concerns about the quality of GxE literature are growing due to low replicability^4^,^39^. The expansion of candidate GxE studies has given way to a literature inundated by novel findings with small effect sizes and its disappointing replications^39^. Many variables, definitions, and analyses have been generated by the large number of testable GxE hypotheses, increasing the risk that only significant results are published. For that reason, a strong publication bias in direction of novel positive GxE findings turns candidate findings more robust than they really are. Moreover, replication of candidate GxE studies seem also to be biased toward positive results because positive replication investigations have smaller sample sizes on average than negative ones^4^. However, our results corroborate previous findings that suggest a significant effect of maternal smoking during pregnancy in offspring externalizing behaviors even though genetic effects could also influence these behaviors^27^. It is important to highlight that further research on this topic is essential for world public health^40^,^41^.

The heterogeneity of results in GxE research may be explained by methodological differences, for instance, in terms of sample composition, design, and outcome measures^9^. As such, there are many statistical considerations and issues about both how suitable genetic and environmental choices should be done and these choices affect replicability^39^. Failure to correctly control for potential confounders can also be problematic in candidate GxE studies^39^. Furthermore, these studies require appropriate understanding of genetic mechanisms and suitable measurement of the target environment, as well as an integration of these two variables with respect to a specific outcome of interest^39^.

This work should be interpreted in the context of its limitations. First, regarding the validity of self-report data, it is questionable what proportion of pregnant women reports their true smoking status, and whether there is risk of significant underestimation. However, results from the Pelotas cohort in Brazil and the Avon Longitudinal Study of Parents and Children in Britain showed that maternal prenatal smoking in the Brazilian city was reported twice as frequently as in the British Cohort^42^. Additionally, in the Pelotas 1993 Birth Cohort Study, intrauterine exposure to tobacco was previously associated with low birth weight and other outcomes in infancy for which smoking is a well-known risk factor^42^,^43^. Second, the cumulative polygenic effect of other dopaminergic genes was not considered^44^. *COMT* variants could affect antisocial behavior in concert with other genes which act in dopaminergic and serotonergic pathways. Lastly, although retention rates of follow-ups are substantial, another limitation of the present study is the potential bias because of refusals and losses.

Finally, 90% of all individuals under the age of 18 years live in LMIC and many known environmental risk factors for mental disorders tend to be present more frequently in this context. Therefore, the attempt to replicate studies of GxE in LMIC provides a different context for testing the robustness of models of the etiology of mental health problems. Maternal tobacco use during gestation was associated with higher levels of conduct problems at ages 11 and 15 and crimes committed in late adolescence in the present study. However, any significant effect of *COMT* gene or of its interaction with prenatal maternal smoking in conduct problems and criminal offenses were not observed.

## Methods

### Ethics Statement

This project was approved by the Institutional Review Board of the School of Medicine, Federal University of Pelotas. All procedures were performed in accordance with the Declaration of Helsinki for medical research involving human subjects. Parents or legal guardians signed an informed consent form authorizing their own participation and that of the children in the study.

### Subjects

The present study analyzed data from the Pelotas 1993 Birth Cohort Study. A detailed description of characteristics and study design of this sample may be found elsewhere^45^,^46^. Briefly, all children born in 1993 in the city of Pelotas, Brazil, were included in the study (N = 5,265), except 16 mothers who could not be interviewed or refused to participate. Follow-up visits were made at multiple time points. The current study used data collected at the perinatal assessment and follow-ups at ages 11, 15, and 18/19 years, which had retention rates of 87.5%, 85.7% and 81.3%, respectively^47^.

### Phenotypic assessment

At perinatal assessment, mothers were interviewed about a variety of topics, including smoking during pregnancy. The mothers were classified as smokers if they reported having smoked in any trimester of gestation. Data about skin color and child gender, maternal education (number of years attending school grouped in three strata), alcohol use during pregnancy (yes/no), birth weight of newborn and monthly family income (measured in number of minimum wages, a standard unit in Brazil valued around USD 60 in 1993) were collected in the same perinatal assessment^45^,^46^.

At the age 11 visit (in 2004), the validated Brazilian Portuguese version of the Strengths and Difficulties Questionnaire (SDQ) was used to collect child mental health data^48^,^49^. The SDQ conduct subscale (ranging from 0 to 10), answered by the primary caregiver, was used as the outcome measure in the present study. Maternal mental health data were obtained using the Brazilian Portuguese validated version of the Self-Report Questionnaire (SRQ)^50^. At age 15 visit (in 2008), the same strategy was performed to obtain information on conduct problems of participants.

During the age 18/19 assessment (2011/2012), 3,618 adolescents completed a Brazilian Portuguese version of a confidential self-report questionnaire about criminal behavior, originally developed in the Edinburgh Study of Youth Transitions and Crime^51^. This questionnaire included thirteen questions referring to crimes committed by the adolescents in the previous 12 months. A detailed description of these questions and how they were translated for use in the local context can be found elsewhere^3^. We used a count variable as an outcome in the current analyses: the number of types of crimes committed by the youth in the previous 12 months (ranging from X to Y). We also used a categorical variable to complement this analysis, coded positive if the participant reported at least one criminal behavior in the last year.

### DNA collection and genotyping

DNA samples were collected from saliva using the Oragene OG-250 DNA Self-Collection kit (DNA Genotek Inc., Kanata, Ontario, CA) at 15 age visit and were included in the present study (N = 4,101). The *COMT Val*^*158*^*Met* polymorphism was genotyped by TaqMan^®^ allelic discrimination system, following the manufacturer’s recommended protocol (Applied Biosystems Inc., Foster City, CA, USA).

### Statistical analyses

Negative binomial regression analyses were used to examine whether *Val*^*158*^*Met* genotypes interact with prenatal maternal smoking in relation to SDQ conduct measures (at ages 11 and 15) and to criminal behavior, using the number of different types of crimes committed at 18/19 as the outcome variable. Logistic regression, using a categorical variable of any crime committed in the previous 12 months as an outcome, was also used to complement this analysis. Pre-defined potential confounders were analyzed: gender, skin color, family income, maternal prenatal use of alcohol, maternal education and maternal mental health. To test the possible confounders, χ^2^ test was used for categorical variables and Mann-Whitney *U*-test for continuous variables. Covariates were included in the models if they were associated with the study factor and outcome at p ≤ 0.10.

## Additional Information

**How to cite this article**: Salatino-Oliveira, A. *et al*. *COMT* and prenatal maternal smoking in associations with conduct problems and crime: the Pelotas 1993 birth cohort study. *Sci. Rep.* **6**, 29900; doi: 10.1038/srep29900 (2016).

## Supplementary Material

Supplementary Information

## Figures and Tables

**Table 1:** Demographic and clinical characteristics according to genotype groups.

	*COMT Val*^*158*^*Met* genotypes		
	*Val/Val* (N = 1375)	*Val/Met* (N = 1948)	*Met/Met* (N = 772)
Male	48.5%	49.6%	47.8%
White skin color	58.5%	64.0%	72.3%
Family income (minimum wages)	4.04 (6.138)	4.27 (5.654)	4.45 (5.254)
Maternal education (years of school)			
0–4	26.0%	25.5%	23.1%
5–8	43.6%	43.4%	44.4%
9 or more	30.4%	31.1%	32.5%
Maternal alcohol	5.3%	5.1%	5.1%
Maternal smoking	34.8%	32.4%	32.4%
Maternal psychopathology	31.8%	31.5%	27.2%
SDQ conduct (age 11)	2.59 (2.389)	2.49 (2.323)	2.35 (2.175)
SDQ conduct (age 15)	2.32 (2.255)	2.33 (2.282)	2.14 (2.168)
Any crime (age 18)	17.3%	17.1%	18.9%

Categorical data presented as percentages; continuous variables as mean (standard deviation); *COMT*: Cathecol-O-methyltransferase; SDQ: Strengths and Difficulties Questionnaire.

**Table 2:** Analyses of main effects of maternal smoking during pregnancy and *COMT* genotypes in SDQ conduct subscale (at ages 11 and 15) and crime (at age 18), using negative binomial regression analyses.

Variables	SDQ conduct subscale^1^								Criminal offenses^2^			
	At age 11				At age 15				At age 18			
	IRR	95% CI	*χ*^2^	*P*	IRR	95% CI	χ^2^	*P*	IRR	95% CI	χ^2^	*P*
Maternal Smoking												
No	1	—	28.106	**<0.001**	1	—	46.400	**<0.001**	1	—	9.595	**0.002**
Yes	1.24	1.14–1.34			1.32	1.22–1.43			1.28	1.09–1.48		
*COMT* genotypes												
*Met*/*Met*	1	—	0.140	0.932	1	—	1.503	0.472	1	—	2.530	0.282
*Met*/*Val*	1.01	0.92–1.12			1.06	0.95–1.17			0.87	0.71–1.05		
*Val*/*Val*	1.02	0.92–1.14			1.01	0.91–1.13			0.95	0.78–1.17		

SDQ: Strengths and Difficulties Questionnaire; *COMT*: Cathecol-O-methyltransferase gene; IRR: Incidence-rate ratio; 95% CI: 95% confidence interval; MS: Maternal smoking during pregnancy.

^1^Gender, skin color, family income, and maternal mental health variables were included in the analyses (p < 0.020 for all covariables).

^2^Gender and maternal mental health variables were included in the analysis (p < 0.001 for both).

## References

[b1] Association A. P. Diagnostic and statistical manual of mental disorders - Fifth edition (American Psychiatric Publishing, 2013).

[b2] Moffitt T. E. Adolescence-limited and life-course-persistent antisocial behavior: a developmental taxonomy. Psychol Rev. 100, 674–701 (1993). 8255953

[b3] Murray J. . Childhood behaviour problems predict crime and violence in late adolescence: Brazilian and British birth cohort studies. Soc Psychiatry Psychiatr Epidemiol. 50, 579–589 (2015). 25319112 10.1007/s00127-014-0976-z PMC4361758

[b4] Duncan L. E. & Keller M. C. A critical review of the first 10 years of candidate gene-by-environment interaction research in psychiatry. Am J Psychiatry 168, 1041–1049 (2011). 21890791 10.1176/appi.ajp.2011.11020191 PMC3222234

[b5] Caspi A. . Role of genotype in the cycle of violence in maltreated children. Science 297, 851–854 (2002). 12161658 10.1126/science.1072290

[b6] Caspi A. . Influence of life stress on depression: moderation by a polymorphism in the 5-HTT gene. Science 301, 386–389 (2003). 12869766 10.1126/science.1083968

[b7] Thapar A. . Catechol O-methyltransferase gene variant and birth weight predict early-onset antisocial behavior in children with attention-deficit/hyperactivity disorder. Arch Gen Psychiatry 62, 1275–1278 (2005). 16275815 10.1001/archpsyc.62.11.1275

[b8] Sengupta S. M. . *COMT* Val108/158Met gene variant, birth weight, and conduct disorder in children with ADHD. J Am Acad Child Adolesc Psychiatry 45, 1363–1369 (2006). 17075359 10.1097/01.chi.0000251212.44491.46

[b9] Kieling C. . Gene-environment interaction in externalizing problems among adolescents: evidence from the Pelotas 1993 Birth Cohort Study. J Child Psychol Psychiatry 54, 298–304 (2013). 23215821 10.1111/jcpp.12022 PMC3736152

[b10] Matsumoto M. . Catechol O-methyltransferase (*COMT*) mRNA expression in the dorsolateral prefrontal cortex of patients with schizophrenia. Neuropsychopharmacology 28, 1521–1530 (2003). 12799619 10.1038/sj.npp.1300218

[b11] Diaz-Asper C. M., Weinberger D. R. & Goldberg T. E. Catechol-O-methyltransferase polymorphisms and some implications for cognitive therapeutics. NeuroRx 3, 97–105 (2006). 16490416 10.1016/j.nurx.2005.12.010 PMC3593358

[b12] Chen J. . Functional analysis of genetic variation in catechol-O-methyltransferase (*COMT*): effects on mRNA, protein, and enzyme activity in postmortem human brain. Am J Hum Genet 75, 807–821 (2004). 15457404 10.1086/425589 PMC1182110

[b13] Tunbridge E. M., Harrison P. J. & Weinberger D. R. Catechol-o-methyltransferase, cognition, and psychosis: Val158Met and beyond. Biol Psychiatry 60, 141–151 (2006). 16476412 10.1016/j.biopsych.2005.10.024

[b14] DeLisi M. & Vaughn M. G. Foundation for a temperament-based theory of antisocial behavior and criminal justice system involvement. Journal of Criminal Justice 42, 10–25 (2014).

[b15] Caspi A. . A replicated molecular genetic basis for subtyping antisocial behavior in children with attention-deficit/hyperactivity disorder. Arch Gen Psychiatry 65, 203–210 (2008). 18250258 10.1001/archgenpsychiatry.2007.24

[b16] Langley K., Heron J., O’Donovan M. C., Owen M. J. & Thapar A. Genotype link with extreme antisocial behavior: the contribution of cognitive pathways. Arch Gen Psychiatry 67, 1317–1323 (2010). 21135332 10.1001/archgenpsychiatry.2010.163

[b17] Iofrida C., Palumbo S. & Pellegrini S. Molecular genetics and antisocial behavior: where do we stand? Exp Biol Med (Maywood) 239, 1514–1523 (2014). 24764243 10.1177/1535370214529508

[b18] van Goozen S. H. . Identifying mechanisms that underlie links between *COMT* genotype and aggression in male adolescents with ADHD. J Child Psychol Psychiatry 57, 472–480 (2015). 26395975 10.1111/jcpp.12464 PMC5102627

[b19] Salatino-Oliveira A. . Cathechol-O-methyltransferase Val(158)Met polymorphism is associated with disruptive behavior disorders among children and adolescents with ADHD. J Neural Transm (Vienna) 119, 729–733 (2012). 22270685 10.1007/s00702-012-0766-2

[b20] Jin J. . The divergent impact of *COMT* Val158Met on executive function in children with and without attention-deficit/hyperactivity disorder. Genes Brain Behav. 15, 271–279 (2016). 26560848 10.1111/gbb.12270

[b21] Fernandez-Castillo N. & Cormand B. Aggressive behavior in humans: Genes and pathways identified through association studies. Am J Med Genet B Neuropsychiatr Genet 9999, 1–21 (2016). 10.1002/ajmg.b.32419 26773414

[b22] Tuvblad C. . Physical and verbal aggressive behavior and *COMT* genotype: Sensitivity to the environment. Am J Med Genet B Neuropsychiatr Genet [Epub ahead of print] (2016). 10.1002/ajmg.b.32430 26888414

[b23] Hygen B. W. . Child exposure to serious life events, *COMT*, and aggression: Testing differential susceptibility theory. Dev Psychol. 51, 1098–1104 (2015). 26053146 10.1037/dev0000020

[b24] Zhang W., Cao C., Wang M., Ji L. & Cao Y. Monoamine Oxidase A (MAOA) and Catechol-O-Methyltransferase (*COMT*) Gene Polymorphisms Interact with Maternal Parenting in Association with Adolescent Reactive Aggression but not Proactive Aggression: Evidence of Differential Susceptibility. J Youth Adolesc. 45, 812–829 (2016). 26932718 10.1007/s10964-016-0442-1

[b25] Brennan P. A. . Interactions between the *COMT* Val108/158Met polymorphism and maternal prenatal smoking predict aggressive behavior outcomes. Biol Psychol. 87, 99–105 (2011). 21356266 10.1016/j.biopsycho.2011.02.013 PMC3081881

[b26] Dolan C. V. . Testing Causal Effects of Maternal Smoking During Pregnancy on Offspring’s Externalizing and Internalizing Behavior. Behav Genet (2015). 10.1007/s10519-015-9738-2 PMC4826626 26324285

[b27] Kuja-Halkola R., D’Onofrio B. M., Iliadou A. N., Langstrom N. & Lichtenstein P. Prenatal smoking exposure and offspring stress coping in late adolescence: no causal link. Int J Epidemiol. 39, 1531–1540 (2010). 20719746 10.1093/ije/dyq133 PMC3031341

[b28] Talati A. & Weissman M. M. In utero smoking exposure warrants further investigation. Arch Gen Psychiatry 67, 1094 (2010). 20921125 10.1001/archgenpsychiatry.2010.119 PMC3950648

[b29] Beaver K. M., DeLisi M. & Vaughn M. G. A biosocial interaction between prenatal exposure to cigarette smoke and family structure in the prediction of psychopathy in adolescence. Psychiatr Q 81, 325–334 (2010). 20559728 10.1007/s11126-010-9141-3

[b30] D’Onofrio B. M. . Familial confounding of the association between maternal smoking during pregnancy and offspring criminality: a population-based study in Sweden. Arch Gen Psychiatry 67, 529–538 (2010). 20439834 10.1001/archgenpsychiatry.2010.33 PMC3644552

[b31] Gaysina D. . Maternal smoking during pregnancy and offspring conduct problems: evidence from 3 independent genetically sensitive research designs. JAMA Psychiatry 70, 956–963 (2013). 23884431 10.1001/jamapsychiatry.2013.127 PMC3828999

[b32] Boutwell B. B. & Beaver K. M. Maternal cigarette smoking during pregnancy and offspring externalizing behavioral problems: a propensity score matching analysis. Int J Environ Res Public Health 7, 146–163 (2010). 20195438 10.3390/ijerph7010146 PMC2819781

[b33] Ellingson J. M., Goodnight J. A., Van Hulle C. A., Waldman I. D. & D’Onofrio B. M. A sibling-comparison study of smoking during pregnancy and childhood psychological traits. Behav Genet 44, 25–35 (2014). 24085497 10.1007/s10519-013-9618-6 PMC3947202

[b34] Maughan B., Taylor A., Caspi A. & Moffitt T. E. Prenatal smoking and early childhood conduct problems: testing genetic and environmental explanations of the association. Arch Gen Psychiatry 61, 836–843 (2004). 15289282 10.1001/archpsyc.61.8.836

[b35] Maughan B., Taylor C., Taylor A., Butler N. & Bynner J. Pregnancy smoking and childhood conduct problems: a causal association? J Child Psychol Psychiatry 42, 1021–1028 (2001). 11806683 10.1111/1469-7610.00800

[b36] Murray J., Anselmi L., Gallo E. A., Fleitlich-Bilyk B. & Bordin I. A. Epidemiology of childhood conduct problems in Brazil: systematic review and meta-analysis. Soc Psychiatry Psychiatr Epidemiol. 48, 1527–1538 (2013). 23644723 10.1007/s00127-013-0695-x PMC3782642

[b37] Murray J., Cerqueira D. R. & Kahn T. Crime and violence in Brazil: Systematic review of time trends, prevalence rates and risk factors. Aggress Violent Behav. 18, 471–483 (2013). 24027422 10.1016/j.avb.2013.07.003 PMC3763365

[b38] Belsky D. W., Suppli N. P. & Israel S. Gene-environment interaction research in psychiatric epidemiology: a framework and implications for study design. Soc Psychiatry Psychiatr Epidemiol. 49, 1525–1529 (2014). 25216778 10.1007/s00127-014-0954-5 PMC4174337

[b39] Dick D. M. . Candidate gene-environment interaction research: reflections and recommendations. Perspect Psychol Sci. 10, 37–59 (2015). 25620996 10.1177/1745691614556682 PMC4302784

[b40] Wagijo M. A., Sheikh A., Duijts L. & Been J. V. Reducing tobacco smoking and smoke exposure to prevent preterm birth and its complications. Paediatr Respir Rev. [Epub ahead of print] (2015). 10.1016/j.prrv.2015.09.002 26482273

[b41] Wakschlag L. S., Pickett K. E., Cook E. Jr., Benowitz N. L. & Leventhal B. L. Maternal smoking during pregnancy and severe antisocial behavior in offspring: a review. Am J Public Health 92, 966–974 (2002). 12036791 10.2105/ajph.92.6.966 PMC1447496

[b42] Brion M. J. . Maternal smoking and child psychological problems: disentangling causal and noncausal effects. Pediatrics 126, e57–e65 (2010). 20587678 10.1542/peds.2009-2754 PMC3605780

[b43] Horta B. L., Victora C. G., Menezes A. M., Halpern R. & Barros F. C. Low birthweight, preterm births and intrauterine growth retardation in relation to maternal smoking. Paediatr Perinat Epidemiol. 11, 140–151 (1997). 9131707 10.1046/j.1365-3016.1997.d01-17.x

[b44] Thibodeau E. L., Cicchetti D. & Rogosch F. A. Child maltreatment, impulsivity, and antisocial behavior in African American children: Moderation effects from a cumulative dopaminergic gene index. Dev Psychopathol. 27, 1621–1636 (2015). 26535948 10.1017/S095457941500098X PMC4786073

[b45] Victora C. G. . Methodological aspects of the 1993 Pelotas (Brazil) Birth Cohort Study. Rev Saude Publica 40, 39–46 (2006). 16410981 10.1590/s0034-89102006000100008 PMC2917767

[b46] Victora C. G. . Cohort profile: the 1993 Pelotas (Brazil) birth cohort study. Int J Epidemiol. 37, 704–709 (2008). 17846051 10.1093/ije/dym177

[b47] Goncalves H. . Cohort profile update: The 1993 Pelotas (Brazil) birth cohort follow-up visits in adolescence. Int J Epidemiol. 43, 1082–1088 (2014). 24729426 10.1093/ije/dyu077 PMC4121560

[b48] Goodman R. The Strengths and Difficulties Questionnaire: a research note. J Child Psychol Psychiatry 38, 581–586 (1997). 9255702 10.1111/j.1469-7610.1997.tb01545.x

[b49] Fleitlich-Bilyk B. & Goodman R. Prevalence of child and adolescent psychiatric disorders in southeast Brazil. J Am Acad Child Adolesc Psychiatry 43, 727–734 (2004). 15167089 10.1097/01.chi.0000120021.14101.ca

[b50] Mari J. J. & Williams P. A validity study of a psychiatric screening questionnaire (SRQ-20) in primary care in the city of Sao Paulo. Br J Psychiatry 148, 23–26 (1986). 3955316 10.1192/bjp.148.1.23

[b51] McAra L. & McVie S. Youth crime and justice: Key messages from the Edinburgh of Youth Transitions and Crime. Criminology and Criminal Justice 10, 179–209 (2010).

