# Supplementary material for: COMT and prenatal maternal smoking in associations with conduct problems and crime: the Pelotas 1993 birth cohort study
Source: Sci Rep. 2016 Jul 18;6:29900. doi: 10.1038/srep29900 (PMC4947962; doi:10.1038/srep29900)
Supplement: Supplementary Information [file srep29900-s1.doc]

***COMT* and prenatal maternal smoking in associations with conduct problems and crime: the Pelotas 1993 birth cohort study.**

Angélica Salatino-Oliveira^1^, Joseph Murray^2,3^, Christian Kieling^4^, Júlia Pasqualini Genro^1^, Guilherme Polanczyk^5,6^, Luciana Anselmi^3^, Fernando Wehrmeister^3^, Fernando C. de Barros^3,7^, Ana Maria Baptista Menezes^3^, Luis Augusto Rohde^4,6^, Mara Helena Hutz^1*^.

^1^ Department of Genetics, Universidade Federal do Rio Grande do Sul, Porto Alegre, Rio Grande do Sul, Brazil;

^2^ Department of Psychiatry, University of Cambridge, United Kingdom;

^3^ Postgraduate Program in Epidemiology, Universidade Federal de Pelotas, Pelotas, Rio Grande do Sul, Brazil;

^4^ Division of Child and Adolescent Psychiatry, Hospital de Clínicas de Porto Alegre, Porto Alegre, Rio Grande do Sul, Brazil;

^5^ Department of Psychiatry, Universidade de São Paulo, São Paulo, Brazil;

^6^ Institute for Developmental Psychiatry for Children and Adolescents; Brazil;

^7^ Graduate Program in Health and Behavior, Universidade Católica de Pelotas, Pelotas, Rio Grande do Sul, Brazil.

**Supplementary Table S1:** Analyses of interaction between maternal smoking during pregnancy and *COMT* genotypes in SDQ conduct subscale (at ages 11 and 15) and types of crimes (at age 18), using negative binomial regression analyses.

|  |  | **SDQ conduct subscale^1^** | | | | | | | | |  | **Criminal offenses^2^** | | | |
| --- | --- | --- | --- | --- | --- | --- | --- | --- | --- | --- | --- | --- | --- | --- | --- |
| **Variables** | | **At age 11** | | | |  | **At age 15** | | | |  | **At age 18** | | | |
|  |  | **IRR** | **95% CI** | ***χ*^2^** | ***P*** |  | **IRR** | **95% CI** | ***χ*^2^** | ***P*** |  | **IRR** | **95% CI** | ***χ*^2^** | ***P*** |
| **Maternal Smoking** | |  |  |  |  |  |  |  |  |  |  |  |  |  |  |
|  | **No** | **1** | **-** | **26.908** | **<0.001** |  | **1** | **-** | **41.302** | **<0.001** |  | **1** | **-** | **10.702** | **0.001** |
|  | **Yes** | **1.30** | **1.08 - 1.56** |  |  |  | **1.32** | **1.10 - 1.58** |  |  |  | **1.59** | **1.14 - 2.24** |  |  |
| ***COMT* genotypes** | |  |  |  |  |  |  |  |  |  |  |  |  |  |  |
|  | ***Met/Met*** | 1 | - | 0.124 | 0.940 |  | 1 | - | 0.917 | 0.632 |  | 1 | - | 2.630 | 0.268 |
|  | ***Met/Val*** | 1.04 | 0.92 - 1.18 |  |  |  | 1.07 | 0.94 - 1.22 |  |  |  | 0.94 | 0.73 - 1.21 |  |  |
|  | ***Val/Val*** | 1.03 | 0.90 - 1.18 |  |  |  | 0.99 | 0.87 - 1.14 |  |  |  | 1.11 | 0.85 - 1.44 |  |  |
| **Interaction terms** | |  |  |  |  |  |  |  |  |  |  |  |  |  |  |
|  | ***Met/Met* x MS** | 1 | - | 0.610 | 0.737 |  | 1 | - | 0.978 | 0.613 |  | 1 | - | 3.262 | 0.196 |
|  | ***Met/Val* x MS** | 0.92 | 0.75 - 1.14 |  |  |  | 0.96 | 0.78 - 1.19 |  |  |  | 0.82 | 0.55 - 1.23 |  |  |
|  | ***Val/Val* x MS** | 0.97 | 0.77 - 1.21 |  |  |  | 1.05 | 0.84 - 1.32 |  |  |  | 0.68 | 0.44 - 1.04 |  |  |

SDQ: Strengths and Difficulties Questionnaire; *COMT*: Cathecol-O-methyltransferase gene; IRR: Incidence-rate ratio; 95% CI: 95% confidence interval; MS: Maternal smoking during pregnancy.

^1^ Gender, skin color, family income, and maternal mental health variables were included in the analyses (p < 0.021 for all covariables);

^2^ Gender and maternal mental health variables were included in the analysis (p < 0.001 for both);
